# Maternal thyroid function and subsequent markers of insulin resistance in pregnant women with polyendocrine metabolic ovarian syndrome

**DOI:** 10.3389/fendo.2026.1884684

**Published:** 2026-10-16

**Authors:** Anastasia Trouva, Eszter Vanky, Michael Alvarsson, Jan Calissendorff, Bjørn Olav Åsvold, Dorina Ujvari, Angelica Lindén Hirschberg

**Affiliations:** 1 Department of Women’s and Children’s Health, Karolinska Institutet, Stockholm, Sweden; 2 Department of Molecular Medicine and Surgery, Karolinska Institutet, Stockholm, Sweden; 3 Centre for Clinical Research Sörmland, Uppsala University, Eskilstuna, Sweden; 4 Department of Clinical and Molecular Medicine, Faculty of Medicine and Health Sciences, Norwegian University of Science and Technology, Trondheim, Norway; 5 Department of Obstetrics and Gynaecology, St. Olavs Hospital, Trondheim University Hospital, Trondheim, Norway; 6 HUNT Center for Molecular and Clinical Epidemiology, Department of Public Health and Nursing, Norwegian University of Science and Technology (NTNU), Trondheim, Norway; 7 HUNT Research Center, Department of Public Health and Nursing, Norwegian University of Science and Technology (NTNU), Levanger, Norway; 8 Department of Endocrinology, Clinic of Medicine, St. Olavs Hospital, Trondheim University Hospital, Trondheim, Norway; 9 Department Of Microbiology, Tumor and Cell Biology, National Pandemic Centre, Centre for Translational Microbiome Research, Karolinska Institutet, Solna, Sweden; 10 Department of Gynecology and Reproductive Medicine, Karolinska University Hospital, Stockholm, Sweden

**Keywords:** glucose, HOMA-IR, insulin, insulin resistance, PCOS (polycystic ovarian syndrome, pregnancy), thyroid function

## Abstract

**Objectives:**

Low maternal free thyroxine (fT4) levels have been associated with markers of insulin resistance and gestational diabetes. Our primary aim was to investigate whether maternal thyroid function was associated with subsequent insulin resistance in pregnant women with polyendocrine metabolic ovary syndrome (PMOS, previously known as PCOS).

**Methods:**

In this *post-hoc* analysis of two randomized controlled trials, we used linear regression analyses to examine associations of thyroid stimulating hormone (TSH) and fT4 with parameters of metabolic profile (pre- and post-load glucose, fasting insulin and homeostatic model assessment of insulin resistance (HOMA-IR) in 284 pregnant women with PMOS who were randomized to metformin or placebo in early pregnancy. Blood samples were drawn at gestational weeks (gw) 5 to12, 19, 32 and 36. An oral glucose tolerance test was performed at inclusion, gw19 and 32.

**Results:**

At baseline, the women had a mean body mass index (BMI) of 28.9 and HOMA-IR 3.2, and none had overt hypothyroidism. We found significant inverse associations between the change in TSH during pregnancy and post-load glucose at gw19, as well as fasting glucose at gw36, i.e. the smaller increase in maternal TSH, the higher subsequent maternal glucose levels. Furthermore, lower maternal fT4 levels were significantly associated with higher subsequent insulin/HOMA-IR levels, and the change in TSH was also inversely associated with HOMA-IR at gw32. All associations were independent of treatment with metformin or placebo.

**Conclusions:**

In this exploratory *post-hoc* analysis, a smaller maternal TSH increase and lower fT4 levels during pregnancy in overweight women with PMOS are associated with higher subsequent glucose and insulin/HOMA-IR levels later in pregnancy.

## Introduction

Thyroid hormones (free thyroxine (fT4) and free triiodothyronine (fT3)) are important regulators of metabolic activity and energy homeostasis, affecting basal metabolic rate, adenosine triphosphate expenditure, and catecholamine responsiveness (1). They also influence glucose metabolism by stimulating hepatic glycogenolysis and gluconeogenesis, enhancing adipose-tissue lipolysis, increasing circulating free fatty acids, and directly modulating pancreatic insulin and glucagon secretion (2). In addition, thyroid hormones centrally regulate hepatic glucose production through a sympathetic pathway from the hypothalamic paraventricular nucleus to the liver (3).

Clinically, these endocrine connections are reflected by the higher prevalence of thyroid dysfunction, particularly hypothyroidism, among patients with both type 1 (T1DM) and type 2 diabetes mellitus (T2DM) (4, 5). However, recent evidence does not support an association between subclinical thyroid dysfunction and diabetes incidence (6).

During pregnancy, thyroid status normally changes, with a slight increase in thyroid stimulating hormone (TSH) from the first trimester to term and a minimal decrease in free thyroid hormone concentrations. Several studies have linked maternal thyroid function to insulin resistance during pregnancy, i.e. fT4 below the normal range, and even declining fT4 within the normal range, have been associated with a less favorable metabolic profile or gestational diabetes mellitus (GDM) (1, 7). This association is well supported in mid-to-late gestation (7–10), and low fT4 in early pregnancy has also been shown to predispose to GDM (11).

Polyendocrine metabolic ovarian syndrome (PMOS), previously known as polycystic ovary syndrome (PCOS) is a common condition among women of reproductive age, characterized by hyperandrogenism, ovulatory dysfunction and polycystic ovarian morphology (12). Insulin resistance, together with hyperandrogenism, is a pathophysiological cornerstone of PMOS and is present in most affected individuals independently of body mass index (BMI) (12, 13).

Women with PMOS have a substantially increased risk of T2DM later in life and meta-analyses report a 3-fold increased risk of GDM, among other gestational complications (14). Metformin is widely prescribed in PMOS to improve insulin sensitivity; however, treatment during pregnancy has not reduced the incidence of GDM, altered gestational age at diagnosis of GDM, lowered fasting or 2-hour glucose levels, or reduced the need for additional insulin (15).

PMOS and thyroid dysfunction are related conditions (16), and thyroid disease is reported to be more common among women with PMOS both during the peripartum period and outside pregnancy (17–20). The two conditions also share several pregnancy complications, including GDM (14, 21). It remains unknown whether longitudinal changes in thyroid hormone status influence parameters of insulin resistance during pregnancy in women with PMOS, or whether metformin modifies these associations.

The primary aim of this exploratory *post-hoc* study was to investigate serum TSH and fT4 longitudinally during pregnancy in association with subsequent pre- and postload glucose, fasting insulin and Homeostatic Model Assessment for Insulin Resistance (HOMA-IR), and to investigate whether metformin treatment during pregnancy modifies these associations. We hypothesized that lower fT4 levels and/or longitudinal changes in TSH and fT4 during pregnancy would be associated with subsequent parameters of insulin resistance in pregnant women with PMOS.

## Materials and methods

### Study design

This study represents a *post-hoc* evaluation of data gathered from two previous clinical trials: the Pilot study and the PregMet study (22, 23). Both original trials utilized a double-blind, placebo-controlled, randomized design and followed nearly identical protocols.

The primary goal of these foundational studies was to determine if metformin could reduce the occurrence of pregnancy-related complications in women diagnosed with PMOS. Participants were assigned to either a placebo group or a metformin group, with treatment commencing during the first trimester and continuing until birth. Daily metformin dosages differed slightly between the two cohorts, with the Pilot group receiving 1700 mg and the PregMet group receiving 2000 mg.

A comprehensive breakdown of the methodologies used in the initial trials is provided in the **Supplementary Material**.

### Study population

The flowchart of eligible women, exclusions, and the final cohort of randomized participants are shown in **Figure 1**. Twenty-five women participated during two pregnancies, i. e. the first pregnancy in the Pilot and the second in the PregMet study (n=8) or twice in the PregMet study (n=17). The second pregnancy of these participants was excluded from our analyses since insulin sensitivity deteriorates for every subsequent pregnancy and multiparity is a significant risk factor for GDM recurrence (24). The final analysis was based on data from 284 singleton pregnancies, following the exclusion of five individuals: four due to miscarriage and one who was incorrectly diagnosed with PMOS (later confirmed as having congenital adrenal hyperplasia). The cohort was divided into a metformin arm (n = 140) and a placebo arm (n = 144). Blood samples were collected at inclusion, gestational week (gw) 19, 32 and 36. All blood samples were stored at -70 °C.

**Figure 1:**
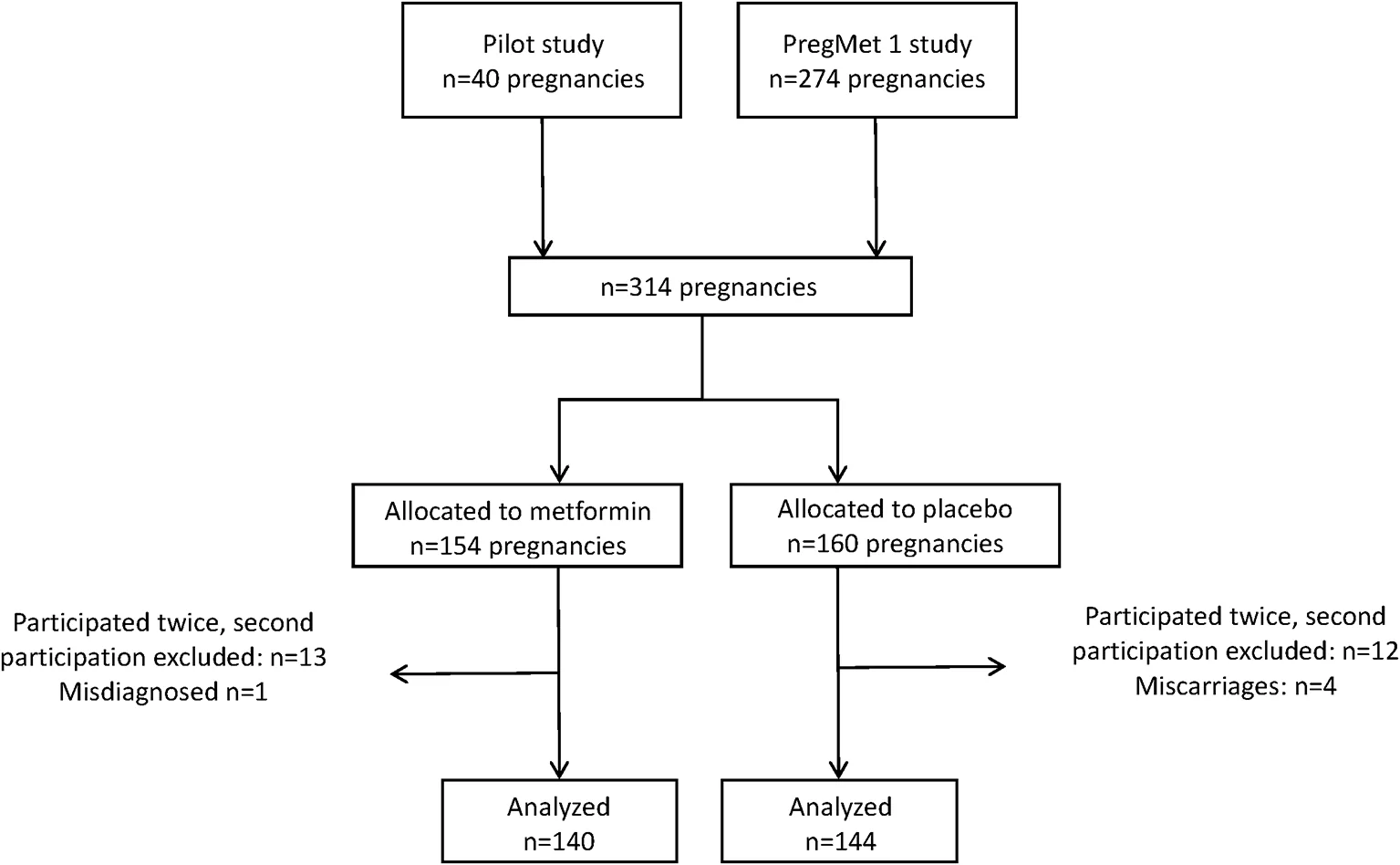
Flowchart of the study design.

Although 14 participants discontinued the study prematurely—five from the metformin group and nine from the placebo group—none rescinded their informed consent. Consequently, all randomized subjects were retained in the dataset. Our evaluation followed the intention-to-treat principle, ensuring that data from all originally assigned participants were utilized in the final results. Five participants required insulin treatment during the trial due to insulin demanding hyperglycemia and for the current study we used their data until the time-point of insulin initiation.

### Laboratory analyses

During the original studies, venous blood samples were drawn from an antecubital vein in the morning after an overnight fast. Blood samples were collected and processed at each study site in accordance with local standardized manual of operation. A 75-g oral glucose tolerance test (OGTT) was performed according to the World Health Organization (1998) recommendations. Glucose was measured during an OGTT (fasting and 120 min post load of 75g glucose) at baseline, gw 19 and 32 as well as fasting at gw 36. Glucose was analyzed as routine analysis at each participating hospital’s laboratory while insulin was determined from serum samples at Trondheim University Hospital by ELISA using kits and reagents supplied- by the manufacturer (DRG Instruments GmbH, Marburg, Germany). The intra- and inter-assay coefficient of variation (CV) was 2.7 and 7.6%, respectively.

TSH and fT4 were measured in samples drawn at baseline, gw 19, 32 and 36. For analysis of thyroid peroxidase antibodies (TPO-ab), we used blood samples collected at inclusion and gw 36. Serum levels of TSH, fT4 and TPO-ab were determined at the Research Laboratory of Women’s and Children’s Health Unit, Karolinska University Hospital, Stockholm, Sweden by chemiluminescent enzyme immunometric assays using commercial kits procured from Siemens Medical Solutions Diagnostics (fT4 and TPO-ab) and Diagnostic Products Corporation (TSH) (Immulite^®^, Los Angeles, CA). TPO-ab was considered positive if the value was ≥ 35 IU/mL. Respective detection limits, intra-assay and inter-assay coefficients of variation were as follows: for TSH 0.004 mIU/L (5.0% and 9.9%), for fT4 3.6 pmol/L (4.5% and 5.5%) and for TPO-ab 7 IU/mL (4.3% and 10.5%). The method-specific reference range provided by the manufacturer was 0.4–4 mIU/L for TSH and 11.5 – 22.7 pmol/L for fT4.

HOMA is a proxy estimate of insulin resistance and beta-cell function derived from basal (fasting) glucose and insulin concentrations (25). We calculated HOMA-IR index using the following formula HOMA-IR= (Fasting Glucose (mmol/L) x Fasting Insulin (mIU/L))/22.5 (26). Although a definitive cutoff value for HOMA-IR has not been established as it can vary based on several factors including ethnicity, age, and gender, a level exceeding 2.9 is commonly associated with insulin resistance. However, a HOMA-IR below 1.5 is indicative of insulin sensitivity, while values between 1.5 and 2.9 suggest impaired insulin sensitivity (25, 27–30). Furthermore, insulin resistance based on HOMA-IR during pregnancy has not been defined.

### Statistical analyses

Continuous variables are expressed as either medians with interquartile ranges or means and standard deviations (SD). For categorical variables, we used frequency counts and percentages.

Repeated measurements of glucose, insulin and HOMA-IR during pregnancy were analyzed using mixed models. These models incorporated treatment group, time point, and their interaction (treatment × time) as primary factors. Where significant interactions were identified, *post-hoc* testing was performed on individual factors while holding the others constant. Multiple comparisons were corrected using Tukey’s adjustment. For the mixed-model analyses, insulin and HOMA-IR were logarithmically transformed because of non-normally distributed values and outliers at certain time points.

Graphical representations of these data include means and 95% confidence intervals (CI) for glucose and geometric means and 95% CIs for insulin and HOMA-IR.

Linear regression was used to examine the associations between metabolic markers (glucose, insulin, and HOMA-IR at gestational weeks 19, 32, and 36) as dependent variables and thyroid function parameters (TSH and fT4 at baseline, gestational weeks 19 and 32, and changes in TSH and fT4 during pregnancy) as independent variables. These regression analyses were performed using the original, untransformed outcome values. To assess whether treatment assignment modified the associations between thyroid function and metabolic outcomes, an interaction term between treatment group and each thyroid function parameter was initially included in the models. As there was no evidence that treatment modified these associations, the interaction term was omitted from the final models, while treatment group was retained as a covariate. Model assumptions were assessed using residual analyses, and Cook’s distance was used to identify influential observations. When influential observations were identified, the models were re-estimated after their exclusion to improve model fit and residual distributions.

Given the large number of associations examined, crude associations with p > 0.10 were not taken forward to adjusted analyses. This threshold was used only to limit number of adjusted models and was not used for confounder selection. For associations taken forward to adjusted analyses, covariates were selected *a priori* based on biological and methodological considerations. BMI was included in all models because of its established association with both thyroid function and insulin resistance. Fasting glucose models were additionally adjusted for age, given its relationship with glucose metabolism during pregnancy. Models with insulin and HOMA-IR as outcomes were additionally adjusted for parity and the parity × treatment interaction, as parity may influence pregnancy-related metabolic adaptation and treatment effects may differ by parity.

For all other analyses, a p-value less than 0.05 was the threshold for statistical significance.

Data processing and calculations were conducted using Statistica 14.0 (TIBCO Software Inc.) and R version 4.2.0. (Copyright (C) 2022 The R Foundation for Statistical Computing).

## Results

**Table 1** summarizes the results of the clinical and metabolic parameters of the participants at baseline, which were similar between the metformin and the placebo groups. The mean BMI of the participants at baseline in both groups was 28.9 and the mean HOMA-IR was 3.2.

**Table 1:** Baseline characteristics of women with PMOS at inclusion, first trimester of pregnancy.

	Total N=284	Metformin N=140	Placebo N=144
Age (years)	29.1 ± 4.4	29.3 ± 4.5	28.8 ± 4.3
Height (cm)	167.5 ± 5.6	167.2 ± 5.8	167.8 ± 5.4
Weight (kg)	80.7 ± 18.8	82.2 ± 19.1	79.2 ± 18.5
BMI (kg/m^2^)	28.9 ± 7.0	29.4 ± 6.6	28.4 ± 7.4
Systolic BP (mm Hg)	118 ± 12	119 ± 13	117 ± 12
Diastolic BP (mm Hg)	73 ± 9	74 ± 9	72 ± 10
Parity n (%)			
P = 0	175 (61.6)	89 (63.6)	86 (59.7)
P≥ 1	109 (38.4)	51 (36.4)	58 (40.3)
Smoking n (%)	19 (6.7)	13 (9.3)	6 (4.2)
Caucasian n (%)	277 (97.9)	134 (96.4)	143 (99.3)
Gestational age at inclusion (days)	72.8 ± 13.9	73.0 ± 14.2	72.7 ± 13.6
Hypothyroidism prior to inclusion n (%)	13 (5.3)	6 (4.9)	7 (5.7)
TPO-ab (+) n (%)	20 (7.2)	10 (7.4)	10 (7.1)
PMOS phenotype			
A (HA+OA+PCOM) n (%)	144 (58.8)	70 (57.4)	74 (60.2)
B (HA+OA) n (%)	9 (3.7)	5 (4.1)	4 (3.2)
C (HA+PCOM) n (%)	25 (10.2)	15 (12.3)	10 (8.1)
D (OA+PCOM) n (%)	67 (27.3)	32 (26.2)	35 (28.5)
Insulin (mIU/L)	15.4 ± 12.0	15.9 ± 12.1	15.0 ± 11.9
HOMA-IR	3.2 ± 2.7	3.3 ± 2.8	3.2 ± 2.7
Fasting glucose (mmol/L)	4.6 ± 0.5	4.6 ± 0.5	4.7 ± 0.6
2-h glucose (mmol/L)	5.6 ± 1.6	5.6 ± 1.5	5.7 ± 1.6
GDM at inclusion n (%)	20 (8.2)	10 (8.2)	10 (8.1)
Metformin use at conception n (%)	88 (30.1)	44 (31.4)	44 (30.6)
Mode of conception			
Spontaneous n (%)	163 (57.4)	82 (58.6)	81 (56.3)
Other n (%)	121 (42.6)	58 (41.4)	63 (43.7)

BMI, Body Mass Index; BP, blood pressure; TPO-ab, thyroid peroxidase antibodies; PMOS, polycystic ovary syndrome; HA, hyperandrogenism; OA, oligo-/anovulation; PCOM, polycystic ovarian morphology; GDM, gestational diabetes mellitus.

Continuous variables are presented as mean ± standard deviations (SD) and categorical variables as number (%) of participants.

At inclusion, 20 women were TPO-antibody positive, with an equal distribution between treatment groups (10 women in each group; **Table 1**). TPO-antibody positivity did not change significantly from inclusion to the end of follow-up. In descriptive comparisons, TPO-antibody positive and TPO-antibody negative women did not differ significantly in glucose, insulin, or HOMA-IR at the assessed gestational time points. TPO-antibody positive women had slightly higher baseline TSH than TPO-antibody negative women, although TSH values remained within the reference range.

The profiles of fasting glucose, insulin and HOMA-IR during pregnancy for the metformin and placebo groups are shown in **Figures 2a–c**. None of the pairwise (metformin/placebo) comparisons regarding glucose, insulin and HOMA-IR at the specific time-points was statistically significant. Regarding the outcome fasting glucose, we found a statistically significant overall time-effect, p<0.0001, independently of treatment (**Figure 2a**). The overall time-effect of insulin (**Figure 2b**) and HOMA-IR (**Figure 2c**) was also statistically significant, p<0.0001, respectively. Furthermore, during the period from baseline to gw 19 the metformin group decreased insulin and HOMA-IR values ​​significantly (p<0.01 and p<0.001 respectively), while the placebo group remained unchanged.

**Figure 2:**
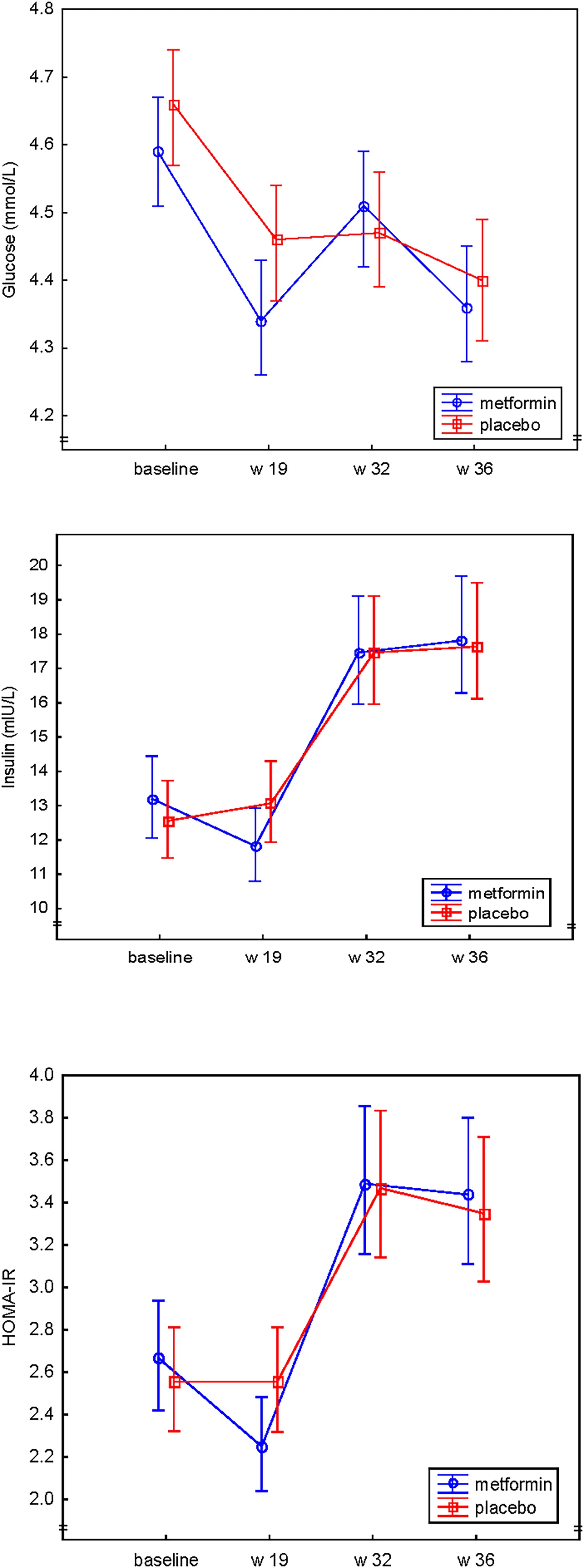
Profiles of fasting glucose, insulin and HOMA-IR during pregnancy for the metformin and placebo groups.

We found that the change (increase) in TSH between a specific timepoint and baseline rather than absolute values of TSH or fT4 was inversely associated with subsequent glucose levels. More precisely, ΔTSH between gw19 and baseline was inversely associated with postload glucose at gw19 (0.24 mmol/L lower glucose for 1 mIU/L higher ΔTSH, p=0.032) (**Figure 3**). Moreover, ΔTSH between gw32 and baseline was inversely associated with fasting glucose at gw36 (0.10 mmol/L lower glucose for 1 mIU/L higher ΔTSH, p=0.020) and so was ΔTSH between gw36 and baseline (0.08 mmol/L lower glucose for 1 mIU/L higher ΔTSH, p=0.037). **Supplementary Table 1** shows the associations between maternal TSH/ΔTSH and fT4/ΔfT4 at the different time-points during pregnancy and subsequent glucose levels.

**Figure 3:**
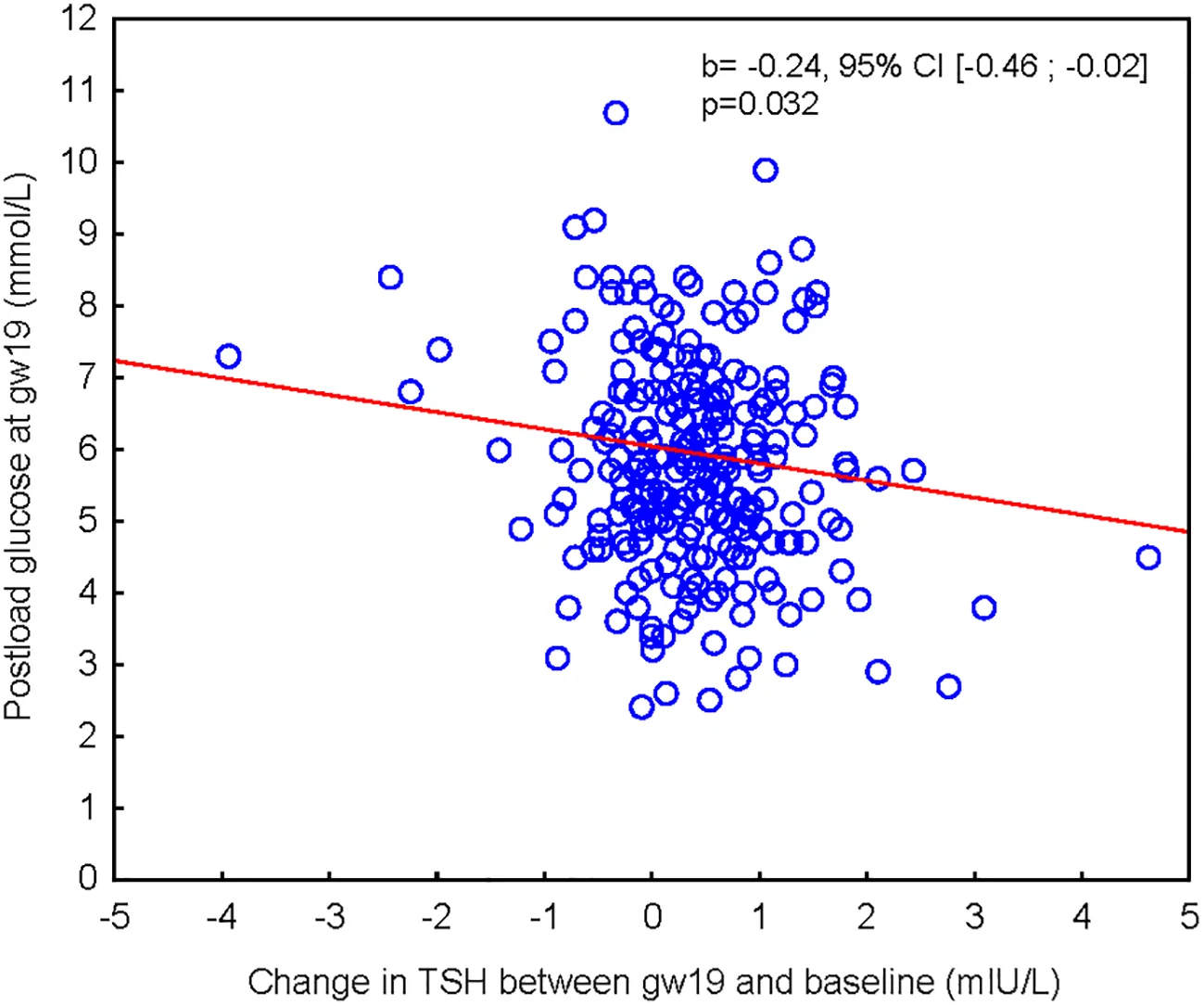
Association between the change in TSH (ΔTSH) between gestational week 19 and baseline and postload glucose levels at gestational week 19. Standardized β= -0.14, 95% CI [-0.26; -0.01]. BMI and treatment were included in the model. Df Residual =236.

We found no direct associations between maternal TSH, ΔTSH or ΔfT4 and insulin levels. However, fT4 at different time-points was negatively associated with insulin levels later in pregnancy. Specifically, fT4 at baseline was inversely associated with insulin at gw19 (0.60 mIU/L lower insulin at gw19 for 1 pmol/L higher fT4 at baseline, p=0.023) and gw36 (1.07 mIU/L lower insulin at gw36 for 1 pmol/L higher fT4 at baseline, p=0.011) (**Figure 4**). Furthermore, fT4 at gw19 was inversely associated with insulin levels at gw36 (1.08 mIU/L lower insulin at gw36 for 1 pmol/L higher fT4 at gw19, p=0.017). Lastly, fT4 at gw32 was negatively associated with insulin at gw36 (1.25 mIU/L lower insulin at gw36 for 1 pmol/L higher fT4 at gw32, p=0.006). **Supplementary Table 2** shows the associations between maternal TSH/ΔTSH and fT4/ΔfT4 at the different time-points during pregnancy and subsequent insulin levels.

**Figure 4:**
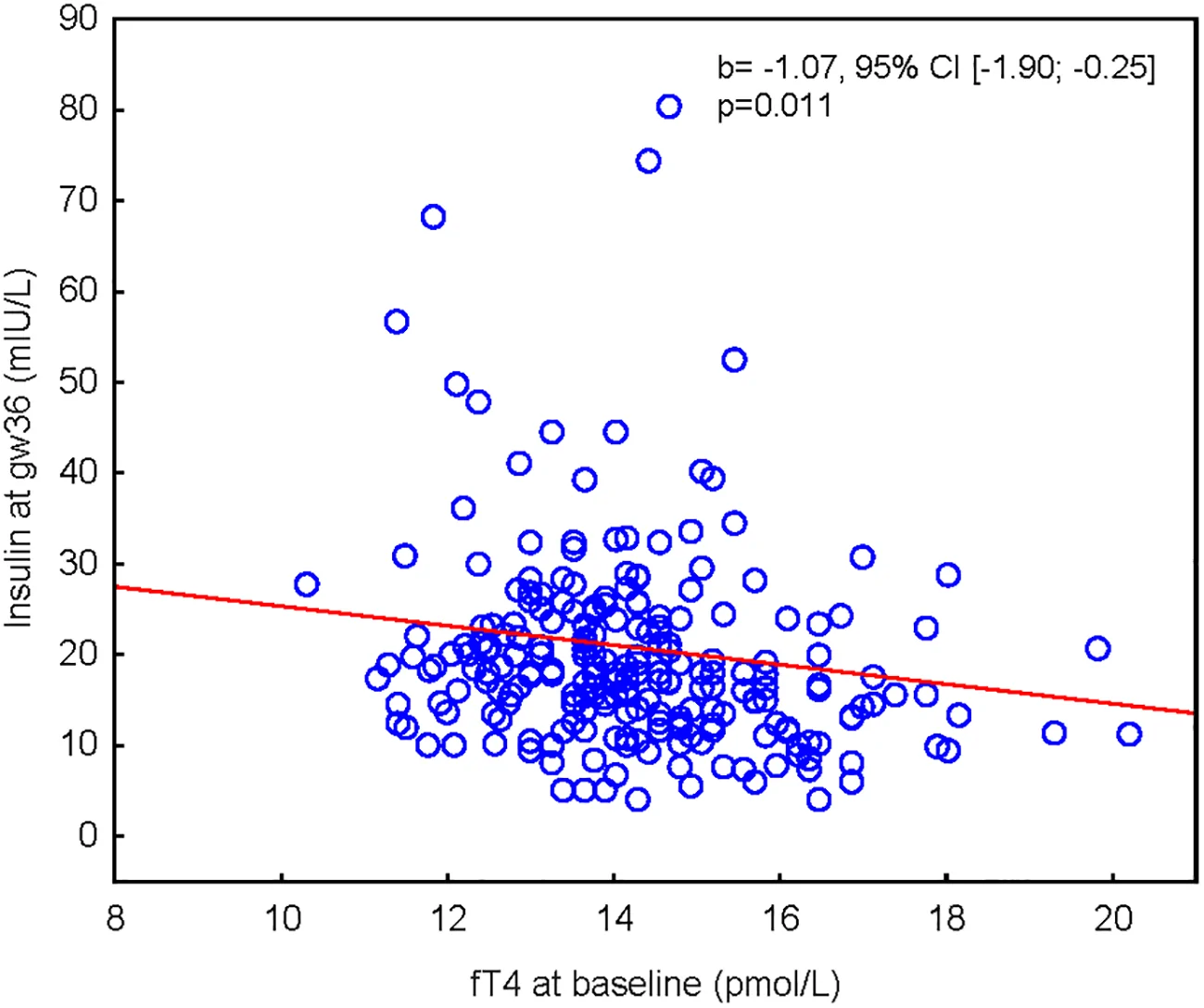
Association between fT4 at baseline and insulin levels at gestational week 36. Standardized β= -0.16, 95% CI [-0.29; -0.04]. BMI, treatment, parity and the interaction parity*treatment were included in the model. Df Residual=223.

In general, the associations of thyroid function parameters with HOMA-IR followed a similar pattern as for insulin. In addition, ΔTSH between gw19 and baseline was inversely associated with HOMA-IR at gw32 (0.34 units lower HOMA-IR for 1 mIU/L higher ΔTSH) (**Figure 5**). FreeT4 at baseline was inversely associated with HOMA-IR at gw19 (0.11 units lower HOMA-IR at gw19 for 1 pmol/L higher fT4 at baseline, p=0.042) and gw36 (0.26 units lower HOMA-IR at gw36 for 1 pmol/L higher fT4 at baseline, p=0.005). Furthermore, fT4 at gw19 was inversely associated with HOMA-IR at gw36 (0.20 units lower HOMA-IR at gw36 for 1 pmol/L higher fT4 at gw19, p=0.024). Lastly, fT4 at gw32 was negatively associated with HOMA-IR at gw36 (0.21 units lower HOMA-IR at gw36 for 1 pmol/L higher fT4 at gw32, p=0.028). **Supplementary Table 3** shows the associations between maternal TSH/ΔTSH and fT4/ΔfT4 at the different time-points during pregnancy and subsequent HOMA-IR levels.

**Figure 5:**
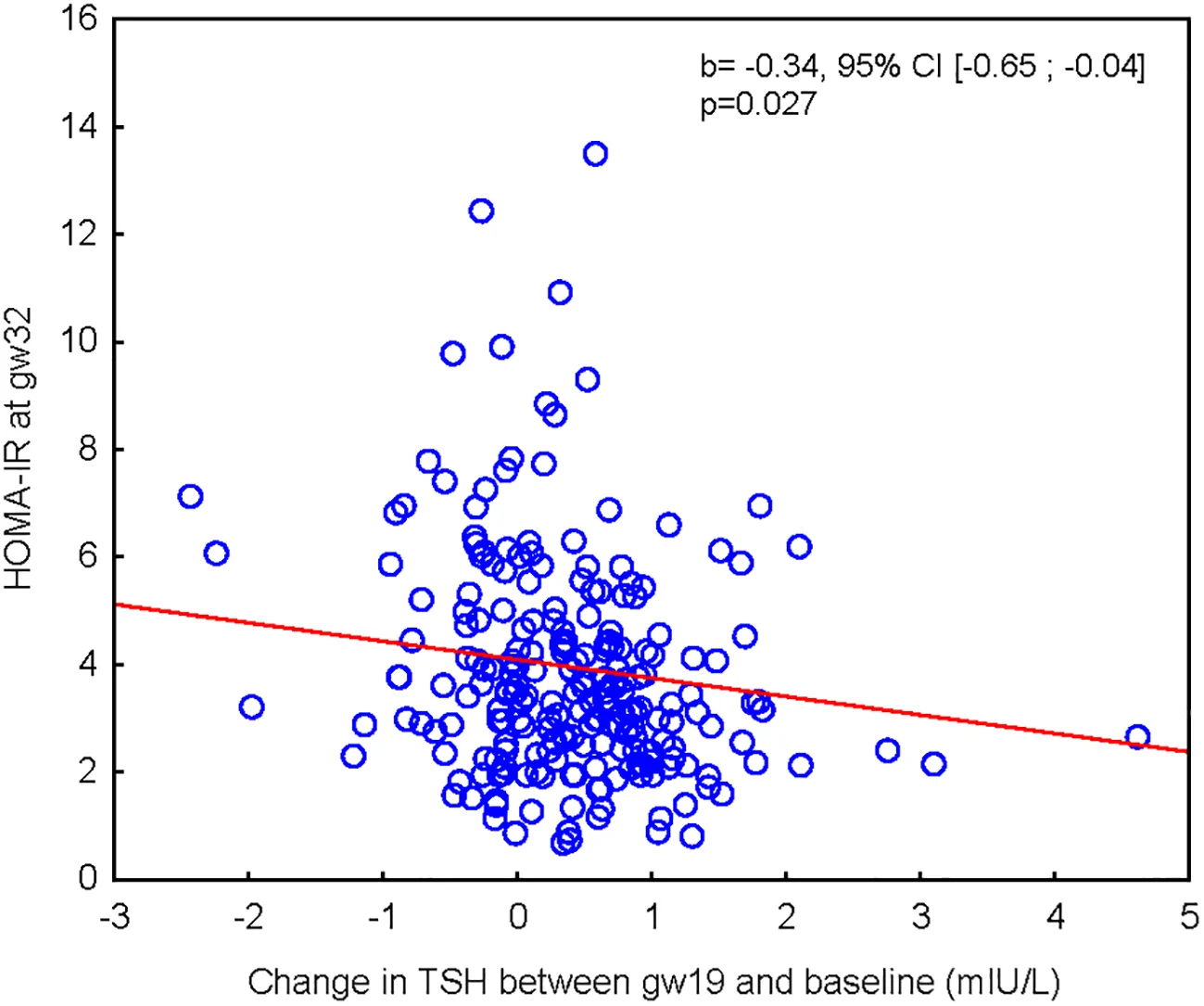
Association between the change in TSH (ΔTSH) between gestational week 19 and baseline and HOMA-IR levels at gestational week 32. Standardized β= -0.13, 95% CI [-0.25; -0.02]. BMI, treatment, parity and the interaction parity*treatment were included in the model. Df Residual=219.

The aforementioned associations were observed independently of treatment group (metformin or placebo).

## Discussion

This exploratory *post-hoc* study examined longitudinal profiles of glucose, insulin and HOMA-IR as well as associations between maternal thyroid function and parameters of insulin resistance among pregnant women with PMOS treated with metformin or placebo. We found that metformin treatment caused significant reductions in insulin and HOMA-IR levels during the early stages of pregnancy, while the placebo group did not. Moreover, there were significant associations between maternal thyroid function and parameters of insulin resistance independently of treatment group, i.e. a relatively smaller increase in TSH during pregnancy was associated with higher subsequent glucose levels, and relatively lower fT4 in early pregnancy was associated with higher insulin and HOMA-IR later in pregnancy.

It is important to note that the participants in our study had a mean BMI of 28.9, placing them at the upper end of the overweight BMI range (BMI 25-29.9), and their mean HOMA-IR index, at 3.2, indicated insulin resistance. Even though the cut-off values used to suggest insulin resistance are not based on a pregnant population, their use may be reasonable during the first trimester of pregnancy. Several studies have reported that early pregnancy HOMA-IR has been associated with subsequent GDM development (31, 32). Thus, HOMA-IR may be regarded as a useful index for estimating insulin resistance during pregnancy. The relatively high mean HOMA-IR level in early pregnancy among the participants in our study may be driven both by PMOS itself and by their BMI level.

There were no significant differences between metformin and placebo on the longitudinal profiles of markers of insulin resistance. However, the initial significant decrease in insulin and HOMA-IR in the metformin group but not in the placebo group may indicate a specific impact of metformin in early pregnancy. Nevertheless, it was unexpected that metformin, a widely used antidiabetic drug which targets insulin resistance, did not affect insulin and glucose levels during pregnancy in a more significant way compared to placebo. This lack of effect has previously been reported in studies of pregnant women with PMOS. In particular, in the meta-analysis of the original studies of our cohort together with the most recent RCT from our research group (15, 23), metformin treatment of women with PMOS during pregnancy had no effect on the development or severity of GDM in this high-risk population, although gestational weight gain was significantly lower in the metformin group (15). Moreover, in most other studies performed in non-PMOS women with overweight/obesity and insulin resistance, no effect of metformin on overall gestational glucose homeostasis has been identified (33–36). One exception is the trial by Chiswick et al. (33), showing that fasting glucose and HOMA-IR at 28 weeks of gestation were significantly lower in women in the metformin group compared to placebo (33). Among women with GDM, metformin appears to reduce gestational weight gain, neonatal hypoglycemia, and macrosomia; however, its effectiveness in achieving glycemic control has not been demonstrated (37). One possible explanation is that the increasing insulin resistance induced by pregnancy itself, which is particularly prominent after mid-pregnancy, cannot be overridden by the effect of metformin.

In a previous study of our cohort, we demonstrated that ΔfT4 between the first and second pregnancy trimesters tended to be inversely associated with GDM (OR 0.85 per 1 pmol/L, 95% CI (0.71;1.02), which means that a relatively more profound decrease in maternal fT4 was associated with GDM (16). This could be explained by the inverse association between fT4 and future insulin and HOMA-IR levels, as we demonstrated in the current study. Furthermore, decreased insulin sensitivity, as determined by increasing HOMA-IR, was associated with decreasing ΔTSH, meaning that a greater increase in TSH between the first and second trimesters was associated with lower HOMA-IR.

The association between low or decreasing levels of maternal fT4 and higher insulin resistance in our patient group is consistent with what has been shown in non-PMOS women. In a study including healthy pregnant women, decreasing fT4 and increasing fT3-to-fT4 ratio in the early third trimester were shown to be associated with higher postload glucose, HbA1c, fasting insulin and HOMA-IR (1). In addition, Cleary-Goldman et al. demonstrated that hypothyroxinemia (normal levels of TSH combined with low levels of fT4) in the second pregnancy trimester was associated with GDM (7). Furthermore, in a case-control study by Oguz et al, mean fT4 levels in the second and third trimesters were significantly lower in the GDM group compared to the control group (9). Similarly, in a study investigating pregnant women with a family history of diabetes, fT4 levels were lower in those who developed GDM compared to those with impaired gestational glucose tolerance, normal glucose tolerance, or healthy pregnant women without family history of diabetes (10). Even though we did not examine associations of maternal thyroid function with the outcome of GDM, our results among women with PMOS are aligned with the conclusion by Yang et al. that low thyroid hormone levels in early pregnancy seem to be a risk factor for GDM incidence (11).

A novel finding in the present study was the association between the change in TSH during pregnancy and measures of insulin resistance, i.e. a smaller increase in TSH was associated with higher glucose and HOMA-IR. A study in non-pregnant women with PMOS demonstrated that a TSH level ≥ 2 mIU/L was associated with insulin resistance, independently of age and BMI (38). The median value of TSH among the women included in our study was < 2 mIU/L at all measured time points. Still, we found that a smaller increase in TSH during pregnancy was associated with higher glucose and HOMA-IR levels.

Several significant associations in the unadjusted models in our study did not remain statistically significant after adjustment for BMI and the actual estimates of association were weakened. This implies that BMI accounts for a considerable degree of the associations between thyroid hormone parameters (both TSH and fT4) and measures of insulin resistance in the studied population. Obesity can induce changes in both TSH and serum thyroid hormones through central and peripheral mechanisms (39).

Whether maternal thyroid parameters provide incremental predictive information beyond established metabolic risk factors, such as BMI and baseline HOMA-IR, remains to be determined in future studies. Thus, the present findings should be interpreted as demonstrating longitudinal associations rather than establishing clinical utility for metabolic risk stratification.

The strengths of our study include the well-characterized population of women with PMOS and the prospective and longitudinal data sampling, allowing us to assess maternal thyroid status throughout pregnancy and study multiple associations between thyroid status and measures of insulin resistance. Although our study is a *post-hoc* analysis of two trials randomizing the participants to metformin and placebo treatment, the current study design allowed us to collect information from all participants combined, accounting for treatment group in the statistical model. One may consider the lack of a control group as a limitation; however, this was beyond the initial scope of our study. Lastly, due to the observational nature of these analyses (the participants were not randomized to higher or lower thyroid function by, for example, levothyroxine treatment), residual confounding may be present.

Another limitation is that glucose was analyzed locally as part of routine clinical analyses at the participating hospitals, whereas insulin and thyroid markers were measured centrally. Although glucose assays are generally more standardized than insulin assays, inter-laboratory variability in local glucose measurements may have introduced some measurement variability in glucose-based outcomes, including HOMA-IR. Conversely, the centralized measurement of insulin reduced inter-assay variability, but insulin remains more susceptible to biological and analytical variability than glucose. This should be considered when interpreting both the glucose-related and insulin/HOMA-IR findings.

A further limitation relates to the interpretation of thyroid function during pregnancy. Population- and trimester-specific reference ranges for TSH and fT4 were not available, and thyroid function was therefore interpreted using the method-specific reference ranges provided by the manufacturer. This may limit the clinical interpretation of absolute thyroid hormone levels during pregnancy, particularly in a study assessing longitudinal thyroid dynamics across gestation. In addition, free thyroid hormone immunoassays may be influenced by pregnancy-related changes in thyroid hormone binding proteins, which could have affected fT4 measurements. However, all thyroid markers were analyzed centrally using the same assay platform, which reduces inter-assay variability in the longitudinal analyses.

Additional limitations include that sensitivity analyses excluding the five participants who required insulin treatment during pregnancy, trial-specific analyses accounting for differences in metformin dose between the Pilot and PregMet cohorts, and adjustment for TPO-antibody status were not performed; therefore, residual effects related to insulin treatment, cohort assignment, or thyroid autoimmunity cannot be entirely excluded, particularly given the small number of TPO-antibody-positive participants.

In summary, this exploratory *post-hoc* analysis suggests that, among pregnant women with PMOS, lower fT4 levels and a smaller TSH increase during pregnancy are associated with higher insulin resistance markers later in pregnancy. These findings should be considered hypothesis-generating and require confirmation in future studies designed to elucidate possible underlying mechanisms for these associations in this specific population.

## Data Availability

The original contributions presented in the study are included in the article/**Supplementary Material**. Further inquiries can be directed to the corresponding author.
