# Supplementary material for: Maternal thyroid function and subsequent markers of insulin resistance in pregnant women with polyendocrine metabolic ovarian syndrome
Source: Front Endocrinol (Lausanne). 2026 Oct 16;17:1884684. doi: 10.3389/fendo.2026.1884684 (PMC13719364; doi:10.3389/fendo.2026.1884684)
Supplement: Supplementary file 1 [file SupplementaryFile1.docx]

**Supplementary material**

**Participant Selection and Criteria**

Eligibility for the Pilot and PregMet 1 trials was based on the Rotterdam criteria. While this was the prospective standard for the PregMet 1 study, the Pilot study—conducted earlier—was evaluated retrospectively to ensure consistency across both cohorts. To be included, participants had to be between 18 and 45 years old, carry a single viable fetus confirmed via ultrasound, and be in their first trimester (gestational weeks 5 through 12).

The researchers excluded individuals with the following:

- Serum creatinine levels exceeding 139 mmol/L or alanine aminotransferase above 90 nmol/L.
- A prior diagnosis of diabetes mellitus or fasting glucose levels exceeding inclusion thresholds.
- History of alcohol abuse, current oral glucocorticoid therapy, or the use of medications that interact with metformin.

**Study Design and Intervention**

Participants were randomized into two groups—metformin or a double-blinded placebo—with stratification based on whether they were using metformin when they conceived. Those using the drug at conception underwent a "wash-out" phase lasting at least seven days prior to the start of the study.

At the outset, all subjects received personalized lifestyle and dietary counseling. The daily regimen consisted of twice-daily doses of either a placebo or metformin (850 mg for the Pilot group and 1000 mg for the PregMet 1 group). Treatment continued until delivery.

**Supplements and Data Collection**

In addition to the study medication, every participant took a daily 1 mg folate supplement and a comprehensive multivitamin (Vitaplex®). The multivitamin provided a broad spectrum of nutrients, including vitamins A, C, D, E, and various B-complex vitamins, along with essential minerals like Iron and Iodine.

Fasting blood samples were collected at four specific intervals:

1. **Baseline:** Gestational weeks 5–12.
2. **Mid-pregnancy:** Week 19 ± 1.
3. **Third Trimester:** Week 32 ± 1.
4. **Pre-delivery:** Week 36 ± 1.

**Compliance**

Adherence to the protocol was high; 80% of the participants self-reported consuming at least 85% of their assigned tablets, meeting the study's threshold for acceptable compliance.
