# Supplementary material for: Maternal thyroid function and subsequent markers of insulin resistance in pregnant women with polyendocrine metabolic ovarian syndrome
Source: Front Endocrinol (Lausanne). 2026 Oct 16;17:1884684. doi: 10.3389/fendo.2026.1884684 (PMC13719364; doi:10.3389/fendo.2026.1884684)
Supplement: Supplementary file 2 [file Table1.docx]

|  | **Glu19-0**  **n=267** | **Glu19-0^a^** | **Glu19-2**  **n=249** | **Glu19-2^a^** | **Glu32-0**  **n=249** | **Glu32-0^a^** | **Glu32-2**  **n=221** | **Glu32-2^a^** | **Glu36**  **n=237** | **Glu36^a^** |
| --- | --- | --- | --- | --- | --- | --- | --- | --- | --- | --- |
| **TSHb**  **n=277** | b=0.02  p=0.564 |  | b= 0.18  p=0.069 | b=0.15  p=0.127 | b=-0.01  p=0.790 |  | b=0.01  p=0.900 |  | b=-0.00  p=0.965 |  |
| **TSH19**  **n=266** |  |  |  |  | b=-0.07  p=0.106 | b=-0.05  p=0.180 | b=0.08  p=0.477 |  | b=0.01  p=0.681 |  |
| **TSH32**  **n=246** |  |  |  |  |  |  |  |  | b=-0.04  p=0.294 |  |
| **fT4b**  **n=276** | b=-0.03  p=0.068 | b=-0.00  p=0.754 | b=0.05  p=0.263 |  | b=-0.04  p=0.070 | b=-0.01  p=0.575 | b=-0.04  p=0.476 |  | b=0.02  p=0.521^*1^  b=-0.06  p=0.024^*2^ | b=0.03  p=0.251^*1^  b=-0.05  p=0.050^*2^ |
| **fT419**  **n=266** |  |  |  |  | b=-0.03  p=0.234 |  | b=-0.06  p=0.346 |  | b=-0.01  p=0.540 |  |
| **fT432**  **n=247** |  |  |  |  |  |  |  |  | b=-0.02  p=0.308 |  |
| **ΔTSH 19-b**  **n=263** | b=-0.06  p=0.103 | b=-0.02  p=0.509 | b=-0.29  p=0.009 | b=-0.24  **p=0.032** | b=-0.06  p=0.146 |  | b=-0.03  p=0.709 |  | b=-0.04  p=0.272 |  |
| **ΔTSH 32-b**  **n=243** |  |  |  |  | b=-0.09  p=0.065 | b=-0.06  p=0.185 | b=-0.03  p=0.772 |  | b=-0.12  p=0.004 | b=-0.10  **p=0.020** |
| **ΔTSH 36-b**  **n=236** |  |  |  |  |  |  |  |  | b=-0.10  p=0.021 | b=-0.08  **p=0.037** |
| **ΔfT4 19-b**  **n=262** | b=0.04  p=0.040 | b=0.02  p=0.226 | b=-0.09  p=0.110 | b=-0.11  p=0.054 | b=0.03  p=0.184 |  | b=-0.01  p=0.843 |  | b=0.03  p=0.282 |  |
| **ΔfT4 32-b**  **n=243** |  |  |  |  | b=0.05  p=0.051 | b=0.03  p=0.193 | b=-0.05  p=0.463 |  | b=0.01  p=0.543 |  |
| **ΔfT4 36-b**  **n=236** |  |  |  |  |  |  |  |  | b=0.04  p=0.050 | b=0.04  p=0.056 |
