# Supplementary material for: Maternal thyroid function and subsequent markers of insulin resistance in pregnant women with polyendocrine metabolic ovarian syndrome
Source: Front Endocrinol (Lausanne). 2026 Oct 16;17:1884684. doi: 10.3389/fendo.2026.1884684 (PMC13719364; doi:10.3389/fendo.2026.1884684)
Supplement: Supplementary file 3 [file Table2.docx]

|  | **Insulin19**  **n=266** | **Insulin19^a^** | **Insulin32**  **n=248** | **Insulin32^a^** | **Insulin36**  **n=240** | **Insulin36^a^** |
| --- | --- | --- | --- | --- | --- | --- |
| **TSHb**  **n=277** | b=0.16  p=0.762 |  | b=0.13  p=0.810 |  | b=-0.08  p=0.913 |  |
| **TSH19**  **n=266** |  |  | b=-0.85  p=0.148 |  | b=-1.00  p=0.272 |  |
| **TSH32**  **n=246** |  |  |  |  | b=-0.08  p=0.929 |  |
|  |  |  |  |  |  |  |
| **fT4b**  **n=276** | b=-1.15  p=0.000 | b=-0.60  **p=0.023** | b=-1.05  p=0.002 | b=-0.47  p=0.110 | b=-1.37  p=0.004 | b=-1.07  **p=0.011** |
| **fT419**  **n=266** |  |  | b=-0.96  p=0.012 | b=-0.65  p=0.057 | b=-1.34  p=0.009 | b=-1.08  **p=0.017** |
| **fT432**  **n=247** |  |  |  |  | b=-1.77  p=0.001 | b=-1.25  **p=0.006** |
|  |  |  |  |  |  |  |
| **ΔTSH19-b**  **n=263** | b=-0.77  p=0.187 |  | b=-2.05  p=0.004 | b=-1.07  p=0.089 | b=-1.78  p=0.051 | b=-0.90  p=0.306 |
| **ΔTSH32-b**  **n=243** |  |  | b=-0.38  p=0.538 |  | b=-1.08  p=0.291 |  |
| **ΔTSH36-b**  **n=236** |  |  |  |  | b=-0.78  p=0.414 |  |
|  |  |  |  |  |  |  |
| **ΔfT419-b**  **n=262** | b=0.59  p=0.089 | b=0.07  p=0.807 | b=0.60  p=0.142 |  | b=0.61  p=0.287 |  |
| **ΔfT432-b**  **n=243** |  |  | b=0.39  p=0.336 |  | b=0.89  p=0.112 |  |
| **ΔfT436-b**  **n=235** |  |  |  |  | b=0.51  p=0.339 |  |
