# Supplementary material for: Maternal thyroid function and subsequent markers of insulin resistance in pregnant women with polyendocrine metabolic ovarian syndrome
Source: Front Endocrinol (Lausanne). 2026 Oct 16;17:1884684. doi: 10.3389/fendo.2026.1884684 (PMC13719364; doi:10.3389/fendo.2026.1884684)
Supplement: Supplementary file 4 [file Table3.docx]

|  | **HOMA19**  **n=261** | **HOMA19^a^** | **HOMA32**  **n=239** | **HOMA32^a^** | **HOMA36**  **n=228** | **HOMA36^a^** |
| --- | --- | --- | --- | --- | --- | --- |
| **TSHb**  **n=277** | b=-0.02  p=0.860 |  | b=0.09  p=0.540 |  | b=0.08  p=0.642 |  |
| **TSH19**  **n=266** |  |  | b=-0.31  p=0.061 | b=-0.26  p=0.078 | b=-0.22  p=0.264 |  |
| **TSH32**  **n=246** |  |  |  |  | b=-0.03  p=0.877 |  |
|  |  |  |  |  |  |  |
| **fT4b**  **n=276** | b=-0.23  p=0.000 | b=-0.11  **p=0.042** | b=-0.22  p=0.005 | b=-0.11  p=0.094 | b=-0.33  p=0.004 | b=-0.26  **p=0.005** |
| **fT419**  **n=266** |  |  | b=-0.18  p=0.026 | b=-0.10  p=0.143 | b=-0.26  p=0.011 | b=-0.20  **p=0.024** |
| **fT432**  **n=247** |  |  |  |  | b=-0.38  p=0.002 | b=-0.21  **p=0.028** |
|  |  |  |  |  |  |  |
| **ΔTSH19-b**  **n=263** | b=-0.14  p=0.271 |  | b=-0.56  p=0.001 | b=-0.34  **p=0.027** | b=-0.39  p=0.043 | b=-0.19  p=0.291 |
| **ΔTSH32-b**  **n=243** |  |  | b=-0.06  p=0.674 |  | b=-0.31  p=0.142 |  |
| **ΔTSH36-b**  **n=236** |  |  |  |  | b=-0.22  p=0.297 |  |
|  |  |  |  |  |  |  |
| **ΔfT419-b**  **n=262** | b=0.14  p=0.051 | b=0.03  p=0.619 | b=0.17  p=0.078 | b=0.02  p=0.829 | b=0.13  p=0.291 |  |
| **ΔfT432-b**  **n=243** |  |  | b=0.11  p=0.248 |  | b=0.13  p=0.266 |  |
| **ΔfT436-b**  **n=235** |  |  |  |  | b=0.10  p=0.366 |  |
